# A Systematic Review of the Usefulness of Glial Fibrillary Acidic Protein for Predicting Acute Intracranial Lesions following Head Trauma

**DOI:** 10.3389/fneur.2017.00652

**Published:** 2017-12-04

**Authors:** Teemu M. Luoto, Rahul Raj, Jussi P. Posti, Andrew J. Gardner, William J. Panenka, Grant L. Iverson

**Affiliations:** ^1^Department of Neurosurgery, Tampere University Hospital, Tampere, Finland; ^2^Department of Neurosurgery, University of Helsinki, Helsinki University Hospital, Helsinki, Finland; ^3^Division of Clinical Neurosciences, Department of Neurosurgery, and Turku Brain Injury Centre, Turku University Hospital, and University of Turku, Turku, Finland; ^4^Priority Research Centre for Stroke and Brain Injury, School of Medicine and Public Health, University of Newcastle, Callaghan, NSW, Australia; ^5^Sports Concussion Program, Hunter New England Local Health District, John Hunter Hospital, New Lambton Heights, NSW, Australia; ^6^British Columbia Neuropsychiatry Program, Department of Psychiatry, University of British Columbia, Vancouver, BC, Canada; ^7^Department of Physical Medicine and Rehabilitation, Harvard Medical School, Spaulding Rehabilitation Hospital, Home Base, A Red Sox Foundation and Massachusetts General Hospital Program, MassGeneral Hospital for Children™ Sports Concussion Program, Boston, MA, United States

**Keywords:** brain injury, head injury, computed tomography, glial fibrillary acidic protein, emergency departments

## Abstract

**Background:**

The extensive use of computed tomography (CT) after acute head injury is costly and carries potential iatrogenic risk. This systematic review examined the usefulness of blood-based glial fibrillary acidic protein (GFAP) for predicting acute trauma-related CT-positive intracranial lesions following head trauma. The main objective was to summarize the current evidence on blood-based GFAP as a potential screening test for acute CT-positive intracranial lesions following head trauma.

**Methods:**

We screened MEDLINE, EMBASE, PsychInfo, CINAHL, Web of Science, the Cochrane Database, Scopus, Clinical Trials, OpenGrey, ResearchGate, and the reference lists of eligible publications for original contributions published between January 1980 and January 2017. Eligibility criteria included: (i) population: human head and brain injuries of all severities and ages; (ii) intervention: blood-based GFAP measurement ≤24 h post-injury; and (iii) outcome: acute traumatic lesion on non-contrast head CT ≤24 h post-injury. Three authors completed the publication screening, data extraction, and quality assessment of eligible articles.

**Results:**

The initial search identified 4,706 articles, with 51 eligible for subsequent full-text assessment. Twenty-seven articles were ultimately included. Twenty-four (89%) studies reported a positive association between GFAP level and acute trauma-related intracranial lesions on head CT. The area under the receiver operating characteristic curve for GFAP prediction of intracranial pathology ranged from 0.74 to 0.98 indicating good to excellent discrimination. GFAP seemed to discriminate mass lesions and diffuse injury, with mass lesions having significantly higher GFAP levels. There was considerable variability between the measured GFAP averages between studies and assays. No well-designed diagnostic studies with specific GFAP cutoff values predictive of acute traumatic intracranial lesions have been published.

**Conclusion:**

Intracranial CT-positive trauma lesions were associated with elevated GFAP levels in the majority of studies. Methodological heterogeneity in GFAP assessments and the lack of well-designed diagnostic studies with commercially validated GFAP platforms hinder the level of evidence, and variability in levels of GFAP with no clearly established cutoff for abnormality limit the clinical usefulness of the biomarker. However, blood-based GFAP holds promise as a means of screening for acute traumatic CT-positive lesion following head trauma.

## Introduction

### Rationale

Since the inception of computed tomography (CT) in the 1970s, its use has increased rapidly (1). Between 1980 and 2017, the number of annual CT scans in the United States has increased from 3 to 62 million (2) with the head being the most commonly imaged area. In modern medicine, non-contrast head CT is the gold standard for identifying significant intracranial injury in an emergency department setting (3). Numerous decision rules [e.g., New Orleans Criteria (4), Canadian CT Head Rule (5)] have been developed in order to focus CT imaging on patients with the greatest risk for clinically significant intracranial injury. However, despite these decision algorithms, a considerable number of trauma head CT scans are performed unnecessarily (1, 6). Almost 80% find no evidence of acute intracranial pathology (7).

There is a non-trivial iatrogenic risk associated with CT scanning, mainly for radiation-induced neoplasia. One head CT significantly increases the risk of subsequent cancer with subsequent CTs conferring additive vulnerability (6, 8). Further, considering the economic cost associated with CT proliferation, judicious use of this imaging modality is important. A growing body of evidence has shown that some blood-based brain trauma biomarkers could aid in predicting which patients will have acute intracranial abnormalities, thus possibly reducing unnecessary head CT scanning (9, 10). Glial fibrillary acidic protein (GFAP) is one of these biomarkers (9, 10).

For several decades, S100B has been investigated as a blood-based marker of brain damage (11). Since the publication of the most recent Scandinavian guidelines for head injury management (12), S100B has been adopted into clinical use mainly in some Nordic countries. According to the guidelines, S100B can be used to substitute head CT scanning in isolated mild head injury patients, who have a low risk for intracranial hemorrhage and are seen within 6 h of injury. Two recent publications have shown that S100B in the context of the Scandinavian guidelines is a safe and cost-effective means of reducing the number of unnecessary CTs in head trauma (13, 14). The clear caveat and applicability-weakening factor of S100B is the sensitivity to extracranial injuries and a short metabolic half-life (11). Theoretically, GFAP is superior to S100B with a more brain injury-specific profile and a longer half-life (15–17).

A substantial body of literature suggests that serum GFAP elevations are associated with acute brain pathology, as evidenced by head CT, across the spectrum of brain injury severity (17–22). Increases in serum levels are detectable within hours of injury and stay elevated for days, a temporal profile that makes GFAP detection potentially very practical and useful in the emergency setting (22, 23).

### Objectives

We conducted a systematic review of the usefulness of blood-based GFAP for predicting acute trauma-related CT-positive intracranial lesions. The main objective was to summarize the current evidence on blood-based GFAP as a potential screening test for acute CT-positive intracranial lesions following head trauma. Our secondary objective was to examine whether or not GFAP was clearly associated with intracranial lesions in patients with mild traumatic brain injury (TBI).

### Research Question

Is increased blood-based GFAP consistently associated with acute (within 24 h post-injury) CT-detectible intracranial trauma lesions following head injury?

## Methods

### Participants

Eligibility criteria included: (i) population: human head and brain injuries of all severities and age groups; (ii) intervention: blood-based GFAP measurement ≤24 h post-injury; and (iii) outcome: acute traumatic lesion on non-contrast head CT ≤24 h post-injury. We focused on emergency management and, therefore, applied the time cutoff of 24 h and examined only blood-based GFAP.

### Systematic Review Protocol

The review was registered with PROSPERO (registration number: CRD42016049452) and adhered to the PRISMA guidelines (24).

### Search Strategy

We screened MEDLINE, EMBASE, PsychInfo, CINAHL, Web of Science, the Cochrane Database, Scopus, Clinical Trials, OpenGrey, ResearchGate, and the reference lists of eligible publications for original contributions published between January 1, 1980, and October 12, 2016. In order to make the review more comprehensive, we updated the literature search on the 10th of February 2017, and thus included original publications published between January 1, 1980 and January 31, 2017. A senior research librarian performed the literature search. The key search terms included: head injur*; head trauma; brain injur*; brain trauma; brain damage; brain contusion*; brain laceration*; brain hemorrhage*; concussion*; MTBI*; TBI*; craniocerebral injur*; craniocerebral trauma; craniocerebral damage; intracranial hemorrhage*; intracranial hematoma; intracranial lesion*; intracranial abnormalit*; intracerebral hemorrhage, traumatic; hematoma, subdural, acute; subdural hematoma; hematoma, epidural, cranial; epidural hematoma; cerebral hemorrhage, traumatic; cerebral hematoma, traumatic; subarachnoid hemorrhage; diffuse axonal injur*; glial fibrillary acidic protein*; astroprotein; glial fibrillary acid protein; glial intermediate filament protein; GFA-protein; GFAP; and GFAP-BDP. The detailed search strategy is presented in an online supplement (Appendix S1 in Supplementary Material). We included only studies published in English.

### Screening and Eligibility

The web-based reference management program Mendeley© (Mendeley Ltd., London, UK) was used for publication screening. Before uploading the references to Mendeley©, duplicate publications (duplicate exclusion: *n* = 2,232; included for screening: *n* = 2,474) were excluded by our librarian. Three authors (Teemu M. Luoto, Rahul Raj, and Jussi P. Posti; hereafter assessing authors) completed the publication screening, data extraction, and quality assessment of eligible articles. Each of the included publications (*n* = 2,474) was initially screened for eligibility based on the title and abstract by two of these assessing authors. After initial screening, 2,423 publications were excluded because they did not fulfill the aforementioned eligibility criteria. Two assessing authors also independently reviewed the full-text versions of a subset of primarily eligible articles (*n* = 51). Conflicts over inclusion were resolved by involving the third assessor.

### Data Extraction and Analysis

Teemu M. Luoto, Rahul Raj, and Jussi P. Posti completed the publication screening, data extraction, and quality assessment of eligible articles. The data extraction form included the following variables: study design and setting, study country and number of sites used, method of GFAP analytics, head CT findings (gradings and percentage of abnormal findings), time intervals between injury and CT/GFAP assessments, extracranial injuries, samples sizes (TBI patient and/or controls), gender and age distributions, GFAP concentrations, and results of relevant statistical tests. All the extracted data were collected on a group level, no individual case level data were available. On an individual article level, two of the assessing authors extracted data independently, and the third reviewed and verified these extraction results. Conflicts over results were resolved by consensus. The scientific quality (including potential sources of bias) of each article was evaluated with the Newcastle–Ottawa Scale (25). The level of evidence was rated according to the Oxford Center of Evidence-based Medicine (26) and GRADE rankings (27). To obtain and confirm missing data (e.g., on study methodology), the investigators of the included publications were contacted by email. Some publications were comprised of overlapping samples, which was acknowledged in the qualitative synthesis. We defined head injury of any severity as the labeling criteria for a case. For example, some studies classified head trauma patients with negative CT scans as “controls.” For this systematic review, these patients were assigned as head trauma cases instead of controls. Results from adult and pediatric studies are reported separately.

## Results

### Study Selection and Characteristics

**Figure 1:**
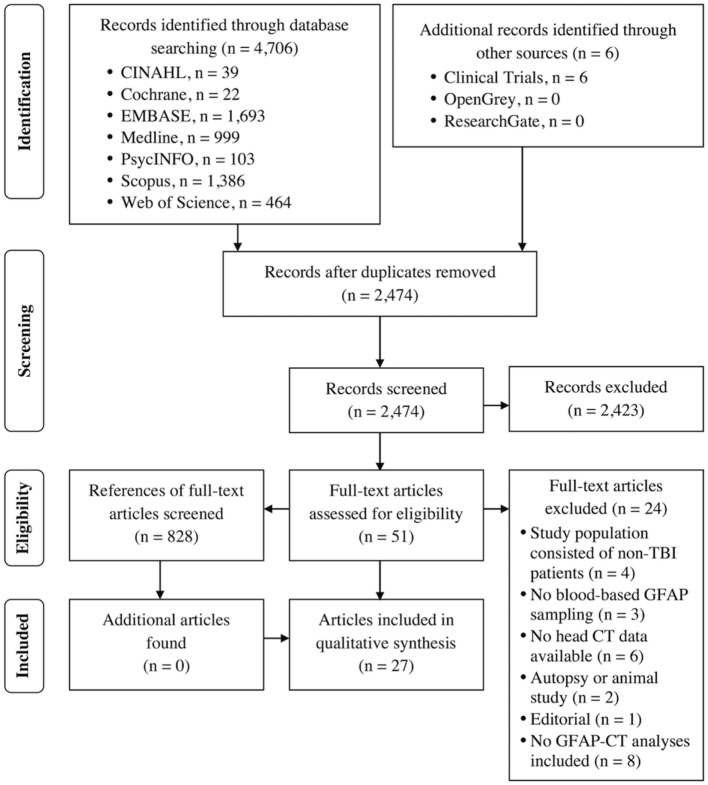
PRISMA flow chart.

The PRISMA flow chart is presented in Figure 1. A total of 27 articles [adult studies: 22 (81%), and pediatric studies: 5 (19%)] were included. Table 1 summarizes the main characteristics of the included studies.

**Table 1:** Summary of study results.

			Controls				TBI								
Reference	Method (GFAP)	Lower level of detection (ng/mL)	*N*, type	Age, years	Male (%)	GFAP level, ng/mL	*N*, severity	Age, years	Male (%)	Time of blood sampling (h after injury)	CT-positive, GFAP level, ng/mL	CT-negative, GFAP level, ng/mL	Acute traumatic lesions on head CT, *n* (%)	GFAP related to lesions	Extracranial injuries accounted for
**Adult studies, mostly MTBI**															

Bogoslovsky et al. (28)	Quanterix Corporation	0.0008	*n* = 69, healthy volunteers	Md = 45, IQR = 31–52	*n* = 35 (51%)	Md = 0.0008, IQR = 0.0008–0.00107	*n* = 34, mild-severe	Md = 39, IQR = 23–52	*n* = 29 (85%)	Admission, ≤22	Md = 0.0176, IQR = 0.00388–0.1296	N/A	*n* = 34 (100%)	Yes	No

	Study design, setting, country, and number of sites; study year(s): case–control; trauma center; USA, eight sites; 2007–2011														
	Main findings: CT-positive TBIs had significantly higher GFAP levels than controls. GFAP was able to discriminate CT-positive TBIs from controls														
	AU-ROC: 0.94 (CT+ vs. controls); cutoff: N/A														

Buonora et al. (29)	Meso Scale discovery	0.21	*n* = 74, healthy volunteers	M = 47, SD = 19	*n* = 15 (30%)	<0.3	*n* = 260, mild-severe	Mild-moderate: M = 47, SD = 19, moderate-severe: M = 47, SD = 21	*n* = 188 (72%)	At admission, no details provided	N/A	N/A	*n* = 132 (51%)	No	Yes

	Study design, setting, country, and number of sites; study year(s): case–control; trauma center; USA, two sites; Canada, four sites; N/A														
	Main findings: no relation between GFAP and CT findings in the cohort with mild-moderate TBI. Higher levels of GFAP in moderate-severe TBI patients compared to controls														
	AU-ROC: N/A; cutoff: N/A														

Diaz-Arrastia et al. (19)^a^	Banyan biomarkers	0.1	*n* = 175, healthy volunteers	M = 34, SD = 14	*n* = 93 (53%)	N/A	*n* = 206, mild-severe	M = 42, SD = 18	*n* = 150 (73%)	M = 10.9, SD = 6.4, min = 0.5, max = 24.3	N/A	N/A	*n* = 106 (51%)	Yes	No

	Study design, setting, country, and number of sites; study year(s): case–control; trauma center; USA, three sites; N/A														
	Main findings: GFAP was able to discriminate: (i) CT-positive TBIs from CT-negative TBIs and (ii) TBIs from controls.														
	AU-ROC: 0.88 (CT+ vs. CT−) and 0.91 (TBI vs. controls); cutoff: N/A														

Honda et al. (15)	BioVendor	0.1	N/A	N/A	N/A	N/A	*n* = 34, mild-severe	CT-positives: Md = 72, IQR = 54–85; CT-negatives: Md = 41, IQR = 30–59	*n* = 22 (65%)	At admission, ≤3	N/A	N/A	*n* = 18 (53%)	Yes	No

	Study design, setting, country, and number of sites; study year(s): cohort; trauma center, Japan, one site; 2006–2007														
	Main findings: GFAP was able to discriminate CT-positive TBIs from CT-negative TBIs.														
	AU-ROC: 0.98 (CT+ vs. CT−); cutoff: N/A														

Lumpkins et al. (30)	BioVendor	N/A	N/A	N/A	N/A	N/A	*n* = 51, mild-severe	M = 43, SD = 21	*n* = 37 (73%)	At admission, no details provided	M = 0.00677, SD = 0.01005	M = 0.00007, SD = 0.00018	*n* = 39 (76%)	Yes	Yes

	Study design, setting, country, and number of sites; study year(s): cohort; trauma center, USA, one site; 2005–2006														
	Main findings: CT-positive TBIs had significantly higher GFAP levels than CT-negative TBIs. GFAP could discriminate CT-positive TBIs from CT-negative TBIs. Also, patients with surgical CT lesions had significantly higher GFAP levels than patients with diffuse lesions.														
	AU-ROC: 0.90 (CT+ vs. CT−); cutoff: 0.001 ng/mL, sensitivity = 62%, specificity = 100%														

McMahon et al. (20)^a^	Banyan Biomarkers	0.01	N/A	N/A	N/A	N/A	*n* = 215, mild-severe	M = 42, SD = 18	*n* = 156 (73%)	At admission, ≤24	M = 2.86, SD = 3.74	M = 0.26, SD = 0.4	*n* = 110 (51%)	Yes	Yes

	Study design, setting, country, and number of sites; study year(s): cohort; trauma center, USA, one site; N/A														
	Main findings: CT-positive TBIs had significantly higher GFAP levels than CT-negative TBIs. GFAP could discriminate CT-positive TBIs from CT-negative TBIs.														
	AU-ROC: 0.87 (CT+ vs. CT−); cutoff: 1.66 ng/mL, sensitivity = 45%, specificity = 99%, Brier score = 0.29														

Metting et al. (31)	BioVendor	0.045	N/A	N/A	N/A	N/A	*n* = 94, mild	M = 34.3, SD = 13.9	N/A	M = 2.4, SD = 2.1	M = 1.20, SD = 1.65	M = 0.05, SD = 0.17	*n* = 19 (20%)	Yes	Yes

	Study design, setting, country, and number of sites; study year(s): cohort; University hospital, The Netherlands, one site; 2005–2007														
	Main Findings: CT-positive TBIs had significantly higher GFAP levels than CT-negative TBIs														
	AU-ROC: N/A; cutoff: N/A														

Okonkwo et al. (18)^a^	Banyan Biomarkers	0.1	N/A	N/A	N/A	N/A	*n* = 215, mild-severe	M = 42, SD = 18	*n* = 157 (73%)	M = 10.9, SD = 6.4, min = 0.5, max = 23.4	M = 2.86, SD = 3.74	M = 0.26, SD = 0.41	*n* = 109 (51%)	Yes	No

	Study design, setting, country, and number of sites; study year(s): cohort; trauma center, USA, three sites; N/A														
	Main Findings: GFAP was able to discriminate CT-positive TBIs from CT-negative TBIs														
	AU-ROC: 0.88 (CT+ vs. CT−); cutoff: 0.68 ng/mL, sensitivity = 73%, specificity = 89%, positive predictive value = 87%														

Papa et al. (16)^d^	Banyan Biomarkers	0.000008	*n* = 188, non-TBI trauma controls	M = 40, SD = 16	*n* = 103 (55%)	N/A	*n* = 209, mild-moderate	M = 40, SD = 16	*n* = 131 (63%)	M = 3.1, 95% CI = 3.0–3.3	N/A	N/A	*n* = 20 (10%)	Yes	Yes

	Study design, setting, country, and number of sites; study year(s): case–control; trauma center, USA, one site; N/A														
	Main Findings: GFAP was able to discriminate CT-positive TBIs from CT-negative TBIs														
	AU-ROC: 0.84 (CT+ vs. CT−); cutoff: 0.067 ng/mL, sensitivity = 100%, specificity = 55%, negative predictive value = 100%, positive predictive value = 20%														

Papa et al. (32)	Banyan Biomarkers	0.02	*n* = 199, no injuries and trauma controls	No injuries: M = 37, SD = 14; trauma controls: M = 44, SD = 17	*n* = 109 (55%) (no injuries: *n* = 93; 53%; trauma controls: *n* = 16; 70%)	M = 0.057, 95% CI = 0.044–0.071	*n* = 108, mild-moderate	M = 39, SD = 15	*n* = 70 (65%)	M = 2.6, 95% CI = 2.4–2.9	N/A	N/A	*n* = 32 (30%)	Yes	No

	Study design, setting, country, and number of sites; study year(s): case–control; Trauma center, USA, 3 sites; N/A														
	Main findings: GFAP could discriminate CT-positive TBIs from CT-negative TBIs. GFAP was more reliable in discriminating: (i) TBIs from controls and (ii) TBIs with surgical CT lesions from non-surgical lesions														
	AU-ROC: 0.79 (CT+ vs. CT−) and 0.90 (TBI vs. controls); cutoff: 0.035 ng/mL, sensitivity = 97%, specificity = 18%, negative predictive value = 94%														

Papa et al. (23)	Banyan Biomarkers	0.008	*n* = 259, trauma controls	M = 41, SD = 16, range = 18–83	*n* = 150 (58%)	Md = 0.008, IQR = 0.008–0.030; range = 0.008–0.773	*n* = 325, mild-moderate	M = 39, SD = 16, range = 18–78	*n* = 212 (65%)	M = 3.0, SD = 0.9	Md = 0.588, IQR = 0.140–2.014, range = 0.008–8.078	Md = 0.033, IQR = 0.008–0.189, range = 0.008–7.785	*n* = 35 (11%)	Yes	No

	Study design, setting, country, and number of sites; study year(s): case–control; trauma center, USA, one site; 2010–2004														
	Main findings: CT-positive TBIs had significantly higher GFAP levels than CT-negative TBIs. GFAP could discriminate CT-positive TBIs from CT-negative TBIs														
	AU-ROC: 0.86 (CT+ vs. CT−); cutoff: N/A														

Posti et al. (21)	The Evidence Investigator Cerebral Custom Array IV	N/A	*n* = 81, orthopedic controls	M = 44.9, SD = 18.8	*n* = 35 (43%)	N/A	*n* = 324, mild-severe	M = 45.3, SD = 19.2	*n* = 238 (74%)	At admission, <24	N/A	N/A	*n* = 200 (69%)	Yes	No

	Study design, setting, country, and number of sites; study year(s): cohort; University hospital; Finland, one site; the United Kingdom, one site; 2011–2003														
	Main findings: CT-positive TBIs had significantly higher GFAP levels than CT-negative TBIs. GFAP could discriminate CT-positive TBIs from CT-negative TBIs. Also, GFAP levels were significantly higher in patients with mass lesions than with non-mass lesions														
	AU-ROC: 0.74 (CT+ vs. CT−); cutoff: N/A														

Shehab and Nassar (33)	ELISA assay, not otherwise specified	N/A	*n* = 20, healthy volunteers	N/A	N/A	M = 0.0015, SD = 0.00037	*n* = 70, mild-severe	M = 40.8, SD = 8, range = 22–64	*n* = 52 (74%)	At admission, no details available	M = 0.1029, SD = 0.0471	M = 0.0668, SD = 0.0224	*n* = 43 (61%)	Yes	Yes

	Study design, setting, country, and number of sites; study year(s): case–control; University hospital, Egypt, one site; N/A														
	Main findings: CT-positive TBIs had significantly higher GFAP levels than CT-negative TBIs. Also, TBIs had significantly higher GFAP levels than controls														
	AU-ROC: N/A; cutoff: N/A														

Welch et al. (17)^e^	Banyan Biomarkers	0.02	N/A	N/A	N/A	N/A	*n* = 231, mild-moderate	M = 45.6, SD = 18.4	*n* = 151 (60%)	At admission, ≤6	Md = 0.1105, IQR = 0.0204–0.4318	Md = 0.0078, IQR = 0.0027–0.0221	*n* = 36 (14%)	Yes	No

	Study design, setting, country, and number of sites; study year(s): cohort; trauma center; USA, five sites; Hungary, two sites; N/A														
	Main findings: GFAP could discriminate CT-positive TBIs from CT-negative TBIs														
	AU-ROC: 0.79 (CT+ vs. CT−); cutoff: 0.015 ng/mL, sensitivity = 81%, specificity = 67%														

Welch et al. (22)^e^	Banyan Biomarkers	0.02	N/A	N/A	N/A	N/A	*n* = 167, mild-moderate	M = 46.0, SD = 17.8	*n* = 102 (61%)	Multiple time points: 0–6, >6–12, >12–18, and >18–24 h	Md = 0.122, IQR = 0.020–0.437	Md = 0.010, IQR = 0.004–0.031	*n* = 33 (20%)	Yes	No

	Study design, setting, country, and number of sites; study year(s): cohort; trauma center; USA, five sites; Hungary, two sites; N/A														
	Main findings: GFAP could discriminate CT-positive TBIs from CT-negative TBIs														
	AU-ROC: 0.84–0.94 (CT+ vs. CT−); cutoff: N/A														

**Adult studies, moderate and severe TBI**															

Lei et al. (34)	BioVendor	0.045	*n* = 135, healthy blood donors	M = 39.2, SD = 15.3, range = 18–65	*n* = 88 (65%)	Md = 0, IQR = 0–0, range = 0.048–0.076	*n* = 67, severe	M = 37.2, SD = 14.3	*n* = 51 (76%)	At admission, ≤4	Md = 1.924, IQR = 0.891–3.126	N/A	*n* = 64 (100%)	Yes	No

	Study design, setting, country, and number of sites; study year(s): case–control; trauma center, China, one site; 2011–2004														
	Main findings: TBIs had significantly higher GFAP levels than controls. Also, patients with surgical CT lesions had significantly higher GFAP levels than patients with diffuse lesions														
	AU-ROC: N/A; cutoff: N/A														

Mondello et al. (35)^f^	BioVendor	N/A	*n* = 167, healthy blood donors	M = 36.9, SD = 14.1	*n* = 95 (57%)	M = 0.07, SD = 0.03	*n* = 81, severe	M = 47.9, SD = 20.4	*n* = 65 (80%)	At admission, no details provided	N/A	N/A	*n* = 80 (99%)	Yes	Yes

	Study design, setting, country, and number of sites; study year(s): case–control; trauma center; USA, two sites; Hungary, two sites; N/A														
	Main findings: TBIs had significantly higher GFAP levels than controls. Also, patients with mass lesions on CT had significantly higher GFAP levels than patients with diffuse lesions														
	AU-ROC: N/A; cutoff: N/A														

Mondello et al. (36)^f^	BioVendor	N/A	N/A	N/A	N/A	N/A	*n* = 59, severe	M = 46.7, range = 19–89	*n* = 46 (78%)	M = 9, SEM = 1	N/A	N/A	*n* = 58 (98%)	Yes	No

	Study design, setting, country, and number of sites; study year(s): cohort; trauma center; USA, two sites; Hungary, two sites; N/A														
	Main findings: TBI patients with mass lesions on CT had significantly higher GFAP levels than patients with diffuse lesions. GFAP was able to discriminate TBIs with mass lesions from TBIs with diffuse lesions														
	AU-ROC: 0.72 (mass lesions vs. diffuse lesions); cutoff: N/A														

Pelinka et al. (37)^b^	LIAISON^®^ GFAP and S100B assay	0.03	N/A	N/A	N/A	N/A	*n* = 92, moderate-severe	Md = 39, IQR = 28–55	*n* = 67 (73%)	At admission, <12	N/A	N/A	*n* = 92 (100%)	Yes	No

	Study design, setting, country, and number of sites; study year(s): cohort; trauma center, Austria, three sites; 1999–2002														
	Main Findings: GFAP levels were positively related to the severity of traumatic CT findings (Marshall grade)														
	AU-ROC: N/A; cutoff: N/A														

Pelinka et al. (38)^b^	LIAISON^®^ GFAP and S100B assay	0.03	*n* = 13, polytrauma patients	Md = 39, IQR = 28–48	*n* = 7 (54%)	N/A	*n* = 101, moderate-severe	Md = 39, IQR = 27–55	*n* = 76 (75%)	At admission, <12	N/A	N/A	*n* = 101 (100%)	Yes	No

	Study design, setting, country, and number of sites; study year(s): case–control; trauma center, Austria, three sites; 1999–2003														
	Main findings: GFAP levels were positively related to the severity of traumatic CT findings (Marshall grade)														
	AU-ROC: N/A; cutoff: N/A														

Vos et al. (39)	Future diagnostics	N/A	N/A	N/A	N/A	N/A	*n* = 79, moderate-severe	M = 47.0, range = 18–91	*n* = 57 (72%)	Md = 1, IQR = 0.5–5	Md = 0.1–2.17	Md = 0.02, 95% CI = 0.02–1	*n* = 64 (84%)	Yes	No

	Study design, setting, country, and number of sites; study year(s): cohort; trauma center, The Netherlands, one site; 2004–2006														
	Main findings: GFAP levels were significantly related to the severity of traumatic CT findings (Marshall grade)														
	AU-ROC: N/A; cutoff: N/A														

Vos et al. (40)	ELISA assay, not otherwise specified	N/A	N/A	N/A	N/A	N/A	*n* = 85, severe	Md = 32, range = 15–81	*n* = 61 (72%)	Md = 2.5, range = 0.25–30	N/A	N/A	*n* = 82 (96%)	Yes	No

	Study design, setting, country, and number of sites; study year(s): Cohort; University hospital, The Netherlands, one site, 1999–2000														
	Main findings: GFAP levels were significantly related to the severity of traumatic CT findings (Marshall grade)														
	AU-ROC: N/A; cutoff: N/A														

**Pediatric studies**															

Fraser et al. (41)	Ridascreen Risk Material 10/5	N/A	N/A	N/A	N/A	N/A	*n* = 27, severe	M = 10.6, SD 0.9, range = 2.4–17	*n* = 14 (52%)	At admission, no details available	N/A	N/A	*n* = 27 (100%)	No	Yes

	Study design, setting, country, and number of sites; study year(s): cohort; pediatric intensive care unit, Canada, four sites; N/A														
	Main findings: only severe CT-positive TBI cases included in the study. GFAP failed to correlate with traumatic CT abnormalities														
	AU-ROC: N/A; cutoff: N/A														

Mondello et al. (42)	Meso Scale Discovery	N/A	*n* = 40, patients treated for trivial reason other than head injury	M = 3.9, SD = 3.8	*n* = 23 (58%)	Md = 0.01, IQR = 0.00–0.05	*n* = 45, mild-severe	M = 3.8, SD = 3.8	*n* = 28 (62%)	Md = 4.7, range = 0.5–20.6	Md = 0.73, IQR = 0.15–2.28	Md = 0.21, IQR = 0.08–1.37	*n* = 29 (64%)	Yes	No

	Study design, setting, country, and number of sites; study year(s): case–control; trauma center, USA, one site; N/A														
	Main findings: CT-positive TBIs had significantly higher GFAP levels than controls. GFAP could discriminate mild TBIs from controls. However, GFAP could not discriminate between CT-positive and CT-negative TBIs														
	AU-ROC: 0.81 (mild TBI vs. control); cutoff: N/A														

Zurek and Fedora (43)	BioVendor	N/A	N/A	N/A	N/A	N/A	*n* = 59, severe	M = 8.9	*n* = 36 (61%)	At admission, <3	N/A	N/A	N/A	No	No

	Study design, setting, country, and number of sites; study year(s): cohort; University hospital, The Czech Republic, one site; 2007–2009														
	Main findings: GFAP failed to correlate with traumatic CT abnormalities														
	AU-ROC: N/A; cutoff: N/A														

Papa et al. (44)^c^	Banyan Biomarkers	0.000008	*n* = 60, non-TBI trauma controls	M = 12, SD = 6, range = 0.1–21	*n* = 39 (65%)	Md = 0.03, IQR = 0.01–0.05	*n* = 197, mild-moderate	M = 11.5, SD = 7, range = 0.1–21	*n* = 131 (66%)	M = 3.3, 95% CI = 3.1–3.5	Md = 1.01, 95% CI = 0.59–1.48	Md = 0.18, 95% CI = 0.06–0.47	*n* = 18 (12%)	Yes	No

	Study design, setting, country, and number of sites; study year(s): case–control; trauma center, USA, three sites; N/A														
	Main findings: CT-positive TBIs had significantly higher GFAP levels than CT-negative TBIs. GFAP could discriminate CT-positive TBIs from CT-negative TBIs														
	AU-ROC: 0.82 (CT+ vs. CT−); cutoff: 0.15 ng/mL, sensitivity = 94%, specificity = 47%, negative prediction value = 98%														

Papa et al. (45)^c^	Banyan Biomarkers	0.000008	*n* = 42, non-TBI orthopedic trauma controls	M = 13, SD = 5	*n* = 24 (59%)	Md = 0.03, IQR = 0.01–0.06	*n* = 114, mild-moderate	M = 13, SD = 7	*n* = 76 (67%)	At admission, <6 h	Md = 1.19, IQR = 0.78–5.13	Md = 0.25, IQR = 0.10–0.63	*n* = 8 (9%)	Yes	No

	Study design, setting, country, and number of sites; study year(s): case–control; trauma center, USA, three sites; N/A														
	Main findings: head injury patients had significantly higher GFAP levels than controls. GFAP could discriminate CT-positive TBIs from CT-negative TBIs														
	AU-ROC: 0.85 (CT+ vs. CT−); cutoff: 0.15 ng/mL, sensitivity = 100%, specificity = 36%, likelihood ratio = 1.6														

*M, mean; Md, median; GFAP, glial fibrillary acidic protein; GFAP-BDP, glial fibrillary acidic protein breakdown product; CT, computed tomography; CDE, common data elements; AU-ROC, area under the receiver operating curve; IQR, interquartile range; 95% CI, 95% confidence interval; TBI, traumatic brain injury; CT+, CT-positive (patients with acute traumatic intracranial lesions on head CT); CT−, CT-negative (patients with no acute traumatic intracranial lesions on head CT); cutoff, GFAP cutoff level for a trauma-positive head CT*.

*^a^TRACK-TBI; studies contain some overlapping cases, also GFAP-BDP levels were measured*.

*^b^Mostly the same sample in both studies*.

*^c^Partly the same sample in both studies*.

*^d^GFAP-BDP levels were also measured*.

*^e^Mostly the same sample in both studies*.

*^f^Mostly the same BANDITS sample in both studies*.

*GFAP platforms*:

*1. Quanterix Corporation, Lexington, MA, USA (method:single molecule array)*.

*2. Banyan Biomarkers, Alachua, FL, USA [method: enzyme-linked immunosorbent assay (ELISA)]*.

*3. BioVendor, Brno, Czech Republic; Candler, NC, USA; and Heidelberg, Germany (method: ELISA)*.

*4. The Evidence Investigator Cerebral Custom Array IV Randox Laboratories Ltd., Crumlin, County Antrim, United Kingdom (method: digital immonoassay technology)*.

*5. Meso Scale Discovery, Gaithersburg, MD, USA (method: electro-chemiluminescent immunoassay)*.

*6. LIAISON^®^ GFAP and S100B assay, AB Sangtec Medical, Bromma, Sweden (method: monoclonal immunoluminometric assay)*.

*7. Future diagnostics, Wijchen, the Netherlands (method: 2-site luminometric immunoassay)*.

*8. Ridascreen Risk Material 10/5, R-Biopharm AG, Darmstadt, Germany (method: ELISA)*.

For this review, we re-classified seven (19%) studies as cohort studies although the original authors named those study designs case–control (46). The investigators in 17 (63%) publications did not explicitly report the study design. Of the included studies, 13 (48%) were case–control and 14 (52%) cohort studies. All of the included studies were observational; *none of the studies had a diagnostic test design* (the accuracy of exact GFAP levels in distinguishing CT-positives from CT-negatives). The majority of the studies were conducted in trauma centers in the United States.

### Patient Demographics and Acute Traumatic Lesions

A total of 3,549 participants (68% males) with mild to severe TBI were enrolled in the included studies with individual sample sizes varying between 27 and 325. Control sample sizes varied between 13 and 259 participants, for a sum total of 1,522, of which 54% were males. Orthopedic trauma patients and healthy volunteers were the most commonly enrolled controls; other controls included blood donors and also paid volunteers. The age distribution of the participants was as follows: adult TBI = 15–91 years, pediatric TBI = 0–21 years, adult controls = 18–83 years, and pediatric controls = 0–21 years. Depending on the study, 9–100% of the patients with TBI had acute traumatic lesions on head CT. The Marshall classification (47) was the most commonly used head CT grading system (12 studies, 44%). Many studies reported only gross categories of the traumatic intracranial lesion (i.e., subdural hematoma, contusion, subarachnoid hemorrhage) or only binary CT outcomes (i.e., “positive” or “negative”). A considerable number of studies did not explicitly specify the subtypes of abnormalities that were considered as acute traumatic CT lesions (16 studies, 59%).

### Analytical Platforms

The analytical platforms used to measure GFAP were diverse, with 10 different methods employed across the 27 studies. The sandwich enzyme-linked immunosorbent assay (ELISA) manufactured by Banyan Biomarkers (Banyan Biomarkers, Inc., Alachua, FL, USA) was the most frequently used platform (10 studies, 37%). The second most used (7 studies, 26%) platform was BioVendor (BioVendor, Heidelberg, Germany). In two studies, the precise analytic GFAP platform was not stated (considered as two different individual platforms that are not otherwise specified in this review). The analytic methods are shown in Table 1. Most studies used venous blood as their source for GFAP measurement, although two studies used arterial samples (note: an assumption of venous sampling was made if there was no direct reference to arterial sampling). Four studies out of the 10 that used the Banyan Biomarkers assay also analyzed GFAP breakdown products in addition to native GFAP.

### Synthesized Findings

**Figure 2:**
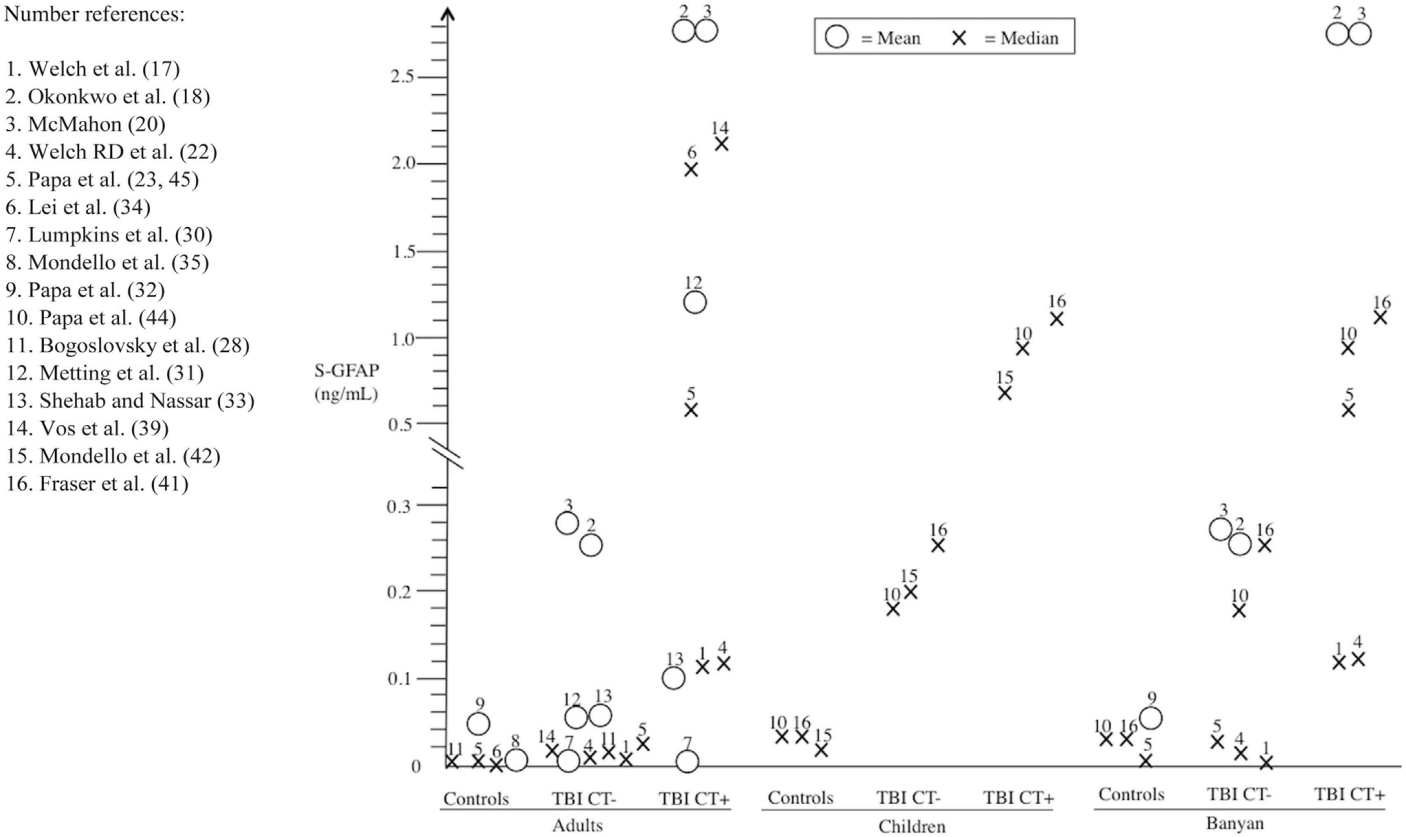
Serum glial fibrillary acidic protein (GFAP) findings (means and medians) from individual studies stratified into different subgroups: (i) controls, (ii) CT-negative traumatic brain injury (TBI) (TBI CT−), and (iii) CT-positive TBIs (TBI CT+). For comparison, the findings are subdivided into adult and pediatric subpopulations, and also results of the most commonly used Banyan Biomarkers (Banyan Biomarkers, Inc., Alachua, FL, USA) assay are presented separately.

There was considerable variability in GFAP levels within the same platform and between platforms (e.g., Banyan Biomarkers vs. BioVendor). For controls (orthopedic injuries and/or healthy participants), the reported GFAP levels varied considerably across studies (adult: range of means = 0.0015–0.057 ng/mL, range of medians = 0–0.0008 ng/mL; and pediatric: range of medians = 0.01–0.03 ng/mL). Between studies, orthopedic controls did not appear to show consistently higher GFAP levels compared to healthy controls. There was only one study that reported results for both non-injured controls and non-TBI trauma controls. In this particular study (32), trauma controls had higher GFAP levels than uninjured control subjects (mean = 0.203, median = 0.216; vs. mean = 0.038, median = 0.010, respectively). The GFAP levels of the TBI patients were consistently higher compared to the controls within studies. Those with CT-positive TBIs (adult: range of means = 0.00677–2.86 ng/mL, range of medians = 0.1–1.9 ng/mL; pediatric: range of medians = 0.73–1.19 ng/mL) had higher GFAP levels than CT-negative cases (adult: range of means = 0.00007–0.26 ng/mL, range of medians = 0.0078–0.33 ng/mL; and pediatric: range of medians = 0.18–1.25 ng/mL). Figure 2 summarizes the mean/median GFAP findings of the individual studies.

Twenty-four (89%) studies reported a positive association between the GFAP level and traumatic lesions seen on head CT. Higher GFAP levels were related to lesion severity in the majority of studies that examined lesion severity (21, 30, 32, 34–36). Additionally, mass lesions and surgically treated lesions were associated with higher GFAP levels than diffuse lesions in all five studies where this comparison was made (21, 30, 32, 35, 36). Thirteen studies reported receiver operating curves on how well GFAP was able to discriminate between head trauma patients with positive vs. negative CT scans. The areas under the receiver operating curves varied between 0.74 and 0.98. Eight studies reported binary classification (CT-positive vs. CT-negative) test results for GFAP (Table 1). Six adult (16–18, 20, 30, 32) and two pediatric (44, 45) studies examined the sensitivity of GFAP cutoff values for identifying intracranial lesions. A pediatric GFAP cutoff value (0.15 mg/mL) was derived from these two studies (44, 45) although it should be noted that the two studies were comprised of a partially overlapping sample. The adult GFAP cutoff values were more inconsistent than the pediatric ones and ranged between 0.001 and 1.66 ng/mL. One adult study (16) established a GFAP cutoff value of 0.067 ng/mL with a sensitivity of 100% and a specificity of 55%.

### Level of Evidence and Risk of Bias

No study was excluded from the review due to a significant source of bias. The Newcastle–Ottawa Scale and the level of evidence (the Oxford Center of Evidence-based Medicine) results are presented in Table 2. The mean level of evidence was 3.6 (17 studies classified as level 4 and 10 studies classified as level 3). On the Newcastle-Ottawa Scale, the average ratings for the 27 studies were as follows: selection (0–4) = 2.9, comparability (0–2) = 1.0, and outcome (0–3) = 3.0. The GRADE ranking for the level of evidence was C. The rating was based on observational studies with fairly consistent results. However, the level of evidence was downgraded, because of partly incomplete data reporting, and the absence of effect estimates.

**Table 2:** The Newcastle–Ottawa Scale scores and the level of evidence of the included studies.

	Newcastle–Ottawa Scale			Center of evidence-based medicine
Reference	Selection (0–4)	Comparability (0–2)	Outcome (0–3)	Level of evidence (1–5)
**Adult**				
Bogoslovsky et al. (28)	⋆⋆⋆	⋆	⋆⋆⋆	4
Buonora et al. (29)	⋆⋆⋆	⋆	⋆⋆	4
Diaz-Arrastia et al. (19)	⋆⋆⋆	⋆⋆	⋆⋆⋆	4
Honda et al. (15)	⋆⋆⋆	⋆	⋆⋆⋆	4
Lei et al. (34)	⋆⋆⋆	⋆	⋆⋆⋆	4
Lumpkins et al. (30)	⋆⋆⋆	⋆⋆	⋆⋆⋆	4
McMahon et al. (20)	⋆⋆⋆	⋆	⋆⋆⋆	3
Mondello et al. (35)	⋆⋆⋆	⋆	⋆⋆⋆	4
Mondello et al. (36)	⋆⋆⋆		⋆⋆⋆	4
Metting et al. (31)	⋆⋆⋆	⋆	⋆⋆⋆	3
Okonkwo et al. (18)	⋆⋆⋆	⋆⋆	⋆⋆⋆	3
Papa et al. (16)	⋆⋆⋆⋆	⋆	⋆⋆⋆	3
Papa et al. (32)	⋆⋆⋆⋆	⋆⋆	⋆⋆⋆	3
Papa et al. (45)	⋆⋆⋆	⋆⋆	⋆⋆⋆	3
Pelinka et al. (37)	⋆⋆		⋆⋆⋆	4
Pelinka et al. (38)	⋆⋆		⋆⋆⋆	4
Posti et al. (21)	⋆⋆⋆		⋆⋆⋆	4
Shehab and Nassar (33)	⋆⋆		⋆⋆⋆	4
Welch et al. (17)	⋆⋆⋆		⋆⋆⋆	4
Welch et al. (22)	⋆⋆⋆⋆	⋆⋆	⋆⋆⋆	3
Vos et al. (39)	⋆⋆⋆		⋆⋆⋆	4
Vos et al. (40)	⋆⋆⋆		⋆⋆⋆	4

**Pediatric**				
Fraser et al. (41)	⋆⋆		⋆⋆⋆	4
Mondello et al. (42)	⋆⋆⋆	⋆⋆	⋆⋆⋆	3
Zurek and Fedora (43)	⋆⋆⋆		⋆⋆⋆	4
Papa et al. (44)	⋆⋆⋆	⋆⋆	⋆⋆⋆	3
Papa et al. (23)	⋆⋆⋆	⋆⋆	⋆⋆⋆	3

### Findings of Studies Examining Mostly Mild TBI

Screening for possible CT-positive trauma-related intracranial lesions is clinically relevant among those with mild head trauma because many of these injuries could be managed without CT imaging. Therefore, the findings of studies examining mostly mild TBI are summarized separately. There were 15 adult studies (15–23, 28–33) and 3 pediatric studies (42, 44, 45) that included *mostly* subjects with mild TBIs, although the samples tended to be heterogeneous and included some patients with moderate or severe TBIs, too. Additionally, the operational criteria for mild TBI were not homogenous among the included studies that examined mostly mild TBIs. This methodological heterogeneity hindered the possibility of organizing and summarizing the results in a combined manner. The research designs were not diagnostic studies of consecutive cohorts of “mild head trauma” cases that employed GFAP as the experimental diagnostic test for intracranial abnormalities compared to a CT or MRI gold standard. Of the included studies, 10 (56%) were case–control and 8 (44%) cohort studies. A total of 2,899 participants were enrolled in the studies with individual study sample sizes varying between 34 and 325 [adults: *n* = 2,543 (88%); pediatric: *n* = 356 (12%)]. Control sample sizes varied between 20 and 259 participants, for a total of 1,207 [adults: *n* = 1,065 (88%); pediatric: *n* = 142 (12%)]. Orthopedic trauma patients and healthy volunteers were the most commonly enrolled controls; other controls included patients treated for trivial reason other than head injury. Six different analytical platforms were employed across the 18 studies. The sandwich ELISA manufactured by Banyan Biomarkers was the most frequently used platform (10 studies, 56%). There was considerable variability in GFAP levels within the same platform, within the same analytic method, and between platforms and this was irrespective of the studied patient and cohort type. Seventeen (94%) of those studies reported a positive association between the GFAP level and the head CT trauma lesions. A considerable number of studies did not explicitly specify the subtypes of abnormalities that were considered as acute traumatic CT lesions (*n* = 6, 33%). Twelve (67%) studies reported receiver operating curves on how well GFAP discriminated CT-positive TBI patients from CT-negative ones. The areas under the receiver operating curves varied between 0.74 and 0.98. Eight (44%) studies reported binary classification (CT-positive vs. CT-negative) test results for GFAP. Six adult (16–18, 20, 30, 32) and two pediatric (44, 45) studies examined GFAP cutoff values for head CT positivity. The mean level of evidence was 3.4 (8 studies classified as level 4 and 10 studies classified as level 3). On the Newcastle–Ottawa Scale, the average ratings for the 18 studies were as follows: selection (0–4) = 3.0, comparability (0–2) = 1.3, and outcome (0–3) = 2.9. The GRADE ranking for the level of evidence was C.

## Discussion

### Summary of Main Findings

Blood levels of GFAP were associated with acute traumatic lesions on head CT. GFAP levels usually were associated with the CT-detectible lesion severity (15–23, 28, 30–40, 42, 44, 45), with surgical lesions (i.e., mass-occupying hematomas/contusions requiring craniotomy) generally showing the highest elevations of serum GFAP (21, 30, 32, 34–36). These findings were consistent across the age spectrum. Based on our review, GFAP holds promise as a potential screening test for acute CT-detectible traumatic brain lesions. However, clearly defined cutoff values (CT-negative vs. CT-positive) for specific GFAP platforms have not been established. The literature has significant methodological limitations that do not allow us to determine the sensitivity or specificity of GFAP for identifying any CT abnormality, or clinically important CT abnormalities, following mild TBI. Well-designed diagnostic test studies for GFAP are needed.

### Findings in Pediatric Samples

Children were the focus of interest in five studies (41–45). Out of these five studies only three investigated serum GFAP levels in relation to CT-negative and CT-positive TBIs (42, 44, 45). In these three pediatric studies, the results were in line with the adult findings. However, the small number of pediatric studies casts some doubt on the generalizability and applicability of these findings. Comparisons between adults and pediatric posttraumatic serum GFAP dynamics have not yet been done to our knowledge. However, normal GFAP levels of healthy children are most likely lower than those of healthy adults in the cerebrospinal fluid (48). In our review, the distribution of GFAP concentrations among adult TBI patients differed somewhat from the pediatric counterparts. The pediatric values were more consistent and the measured range was narrower than with adults (see Table 1; Figure 2). One reason for this is that only two different analytical platforms were used in the positive pediatric studies. In the light of the current evidence, we cannot extrapolate meaningfully as to other factors that account for the difference in adult and pediatric GFAP levels.

### Negative Findings

Three (29, 41, 43) out of the 27 studies did not find any relation between acute (≤24 h post-injury) serum GFAP levels and traumatic head CT findings. Two (41, 43) of these negative studies consisted of pediatric patients with severe TBI. In the first study, Fraser et al. (41) examined whether arterial GFAP was related to traumatic lesions in a sample exclusively consisting of CT-positive severe TBIs. In the second study, Zurek and Fedora (43) compared different Marshall score grades to serum GFAP (Marshall grade distributions not available) and found no relation between GFAP level and Marshall grades. These two negative pediatric studies did not examine GFAP levels in relation to head CT positivity and negativity. They only considered CT-positive cases. Furthermore, among severe TBIs the identification of intracranial traumatic lesions with serum GFAP is not clinically relevant because these patients always require an emergency head CT as part of their routine management. In the only negative adult study, Buonora and co-authors (29) did not find an association between CT-detectible intracranial trauma lesions and GFAP in a case–control study consisting of mild to severe TBIs. The null finding was likely because most of the GFAP levels were below the lower limit of quantification (0.27 ng/mL) and detection (0.21 ng/mL) for their assay. The lower limits of quantification and detection of Buonora’s study were multiple times higher than in other studies.

### Methodological Considerations

We were not able to extrapolate cutoff values or percent increases in GFAP that consistently predict intracranial pathology based on the data presented in the articles. There was considerable variability in measured GFAP levels that was likely related to the analytic GFAP platform employed. Considerable variability in GFAP levels between studies employing the same GFAP platforms was also apparent not only in the TBI groups, but in the orthopedically injured and even within the normal healthy control samples. Time after injury may also be a confounding factor. GFAP was measured from as early as 15 min (40) and as late as to 24 h after TBI within individual studies. GFAP temporal dynamics were examined by only two studies (22, 23). In one study, patients with a positive head CT showed an average GFAP elevation of 3.7% per hour over the first 24 h compared to head trauma cases with negative CTs (22). In the other study, serum GFAP levels were reported to peak 20 h after TBI among those with intracranial lesions detected on CT, and slowly decline over 72 h following injury (23). In most of the included studies, a detailed methodology of sample processing was not reported. This hinders the ability to compare possible factors affecting the GFAP results. In two studies, blood samples were taken from an arterial line and no specific GFAP levels were reported. Whether arterial and venous GFAP levels are comparable is unknown.

Common Data Elements (CDEs) aid in harmonizing neuroimaging data across studies and sites (49). In the studies included in our review, very few utilized the National Institute of Health’s CDEs and explicitly defined which lesions were designated as traumatic. Half of the studies used the Marshall grading to classify head CT findings (15, 21, 28, 31, 34–40, 43). Overall, the studies did not clearly define which intracranial lesions were ascertained as acute TBI abnormalities. For example, some studies [e.g., Mondello et al. (42)] considered skull fractures as an acute traumatic intracranial finding, whereas other studies excluded these lesions. Along with possible discrepancies in trauma lesion interpretation, the technical details of head CT imaging were poorly described. The applied slice thickness and image orientations (sagittal, axial, and coronal) were almost universally lacking. Furthermore, the interpreter (e.g., on call radiologist, neuroradiologist, neurosurgeon) of the head CT images was not stated in the majority of studies. As defined in our inclusion criteria, head CT imaging was performed within 24 h after injury in the included studies. In two studies (17, 43), neuroimaging was conducted within 6 h after injury. Hyperacute (initial hours after injury) imaging can result in false negative scans; for example, some contusions do not demarcate well in the first few hours after trauma.

### GFAP in the Context of Other Diseases and Orthopedic Injuries

Glial fibrillary acidic protein elevations are not specific to TBI; other acute, destructive central nervous system lesions will also raise levels—albeit modestly so. In an exploratory study of a broad spectrum of neurological diseases, GFAP levels were very low in most patients (50). Intracerebral hemorrhage, as a result of cerebrovascular disease, is associated with elevated levels of GFAP (51, 52). Furthermore, high-grade infiltrating tumors (53, 54) and demyelinating diseases [e.g., multiple sclerosis (55)] have also been shown to result in increased serum GFAP. Chronic neurological conditions (e.g., migraines and epilepsy), however, do not appear to influence GFAP levels to a degree that would be expected to confound the usefulness of GFAP as a TBI biomarker (although there are few studies relating to chronic conditions) (50).

It has been reported that injuries outside the brain can also elevate serum GFAP concentrations (56). GFAP has been detected in non-glial and non-central nervous system cells, such as Schwann cells, chondrocytes, fibroblasts, myoepithelial cells, lymphocytes, and liver stellate cells. These can be a source of released GFAP after extremity and bodily trauma. Only a third of the studies accounted for concurrent orthopedic injuries among TBI patients (16, 20, 29–31, 33, 35, 41). The association between GFAP and head CT findings (i.e., CT-positives had higher GFAP levels than CT-negatives of non-head injured controls, and mass lesion were related to higher GFAP levels than diffuse lesions) was present in those studies involving patients with orthopedic injury. Only two adult studies (23, 32) and two pediatric studies (44, 45) reported GFAP levels of trauma controls (median = 0.008, median = 0.03, and median = 0.3 ng/mL, respectively). The pediatric orthopedic control values were derived from mostly the same control cohort. One study (32) examined both non-injured controls and trauma controls; orthopedic trauma seemed to increase acute GFAP levels (non-injured controls: mean = 0.038, 95% CI = 0.029–0.047, median = 0.010, IQR = 0.050; vs. non-TBI trauma controls: mean = 0.203, 95% CI = 0.048–0.357, median = 0.216, IQR = 0.275). To date, this is the only study that directly compares orthopedic injury subjects to non-injured controls. Based on this limited amount of available data, there appears to be an association between head trauma and GFAP levels in studies comparing to orthopedically injured samples, but we cannot draw any solid conclusions on the effects of orthopedic injury on serum GFAP levels.

### Strengths and Limitations

Our review has several strengths. We applied a comprehensive literature search protocol to both the pediatric and adult literature. Gray literature was examined separately. The reference lists of all the eligible articles were screened for potential unidentified new articles. This screening identified no new eligible articles. The literature search protocol can, therefore, be considered inclusive.

There is a potential for publication bias in our conclusions because we only reviewed published articles; studies with negative findings are less likely to be published. Additionally, non-English publications were not included. Most importantly, we could not pool data across studies and meta-analyze GFAP levels. We were unable to conduct a quantitative analysis due to the methodological heterogeneity between studies (e.g., differences in inclusion criteria and reporting of CT findings, variance in GFAP levels among assays), and constraints in the reported principal summary measures (i.e., different measures of central tendency and variance were reported, and no effect sizes were reported). Although measures were taken to gather missing data, the amount of unattainable results was considerable (e.g., GFAP levels of CT-positive cases). The review included articles that utilized partially overlapping patient and/or control samples. Principal investigators of these studies were contacted to clarify the extent of sample overlap. Unfortunately, these investigator inquiries were only partially answered. It was very difficult to determine the association between GFAP levels and CT lesions in patients with mild TBIs because the research designs often included heterogeneous injury severity samples and diverse definitions of injury severity.

Another clinically important limitation of the overall literature is that the sensitivity of the different GFAP assays to epidural hematomas, and the temporal dynamics of GFAP in relation to the evolution of an epidural hematoma, is unknown. Some epidural hematomas, especially in the early stages of evolution, are associated with only mild or modest amounts of parenchymal brain injury—and the extent to which GFAP is elevated in those cases is not known.

### Classification of the Level of Evidence

Based on the classification of the Oxford Center of Evidence-Based Medicine (1 to 5), the level of evidence of the individual studies was 3 or 4 (average = 3.6). The GRADE ranking for the level of evidence is C. A major limitation in most studies was the use of a control group that may not have been representative of the case population. In general, the controls were derived from a completely different population than the cases and limited comparison was performed on the basic demographics (e.g., age, gender, prior health) of these groups (controls vs. TBIs). Case selection was also a point of concern because studies applied widely different inclusion and exclusion criteria. Additionally, very little data (i.e., age, gender, injury mechanism, injury severity, reason for exclusion) was given on the population screened for study inclusion. Thus, the extent to which the results generalize well to a broader population is unknown. The review consisted of studies conducted in multiple countries with different health-care systems. Nevertheless, the main finding that GFAP correlated with CT-detectible intracranial trauma lesions was consistent between these studies. It seems that the results are generalizable and applicable rather globally.

### Future Directions

In the future, larger studies are needed to replicate, extend, and refine these findings. Current ongoing large-scale initiatives, CENTER-TBI (57), and TRACK-TBI (58), are important in verifying the potential of GFAP as a marker in acute TBI triage. From a practical point of view, a rapid capillary blood-based GFAP screening test would be of benefit for patient management in a pre-hospital environment (e.g., sideline assessment in sports and emergency medical services). What is lacking, and clearly needed, is an assay with clearly defined cutoff values for abnormality that has excellent sensitivity and at least good specificity in multiple clinical groups, including those with orthopedic injuries and those with a wide range of pre-existing neurological and medical problems. It is common for people with pre-existing medical, neurological, and neurodegenerative diseases to present to the emergency department with head trauma. Diagnostic studies with clear GFAP cutoffs are needed before possible clinical implementation.

Future well-designed diagnostic studies of GFAP would examine the appropriate spectrum of patients (i.e., the group the test will be applied to in a real-world setting); carefully define the “diagnosis” or condition of interest (e.g., a clinically important lesion on CT, any intracranial lesion on CT, or any traumatic lesion on MRI); apply the neuroimaging to all subjects; use independent or blind comparison with the imaging results; and present sensitivity, specificity, and likelihood ratios (and positive and negative predictive values if the prevalence of intracranial abnormalities in the sample studied is close to the prevalence of intracranial abnormalities in the population of interest). Future researchers should carefully describe their findings in a manner that allows physicians to determine if they can use the results in their work setting, whether the results apply to the patients that they see, and whether the results would actually change clinical practice (e.g., ordering fewer head CT scans). Future researchers are also encouraged to assemble and present case series involving epidural hematomas and carefully examine their GFAP levels and temporal dynamics.

### Conclusion

In conclusion, GFAP is predictive of CT-positive brain damage in acute head injuries. GFAP increases within hours following injury in peripheral blood. A limited number of studies suggest the elevation may peak at 20–24 h post-injury, and thus consideration of temporal dynamics may improve diagnostic sensitivity and specificity. Although promising, at the present time there is not enough evidence to suggest that GFAP can be used clinically as a reliable discriminant of CT-positive and CT-negative brain injury. With future diagnostic research and refinements, GFAP may have the potential to be used as part of a comprehensive diagnostic algorithm to identify patients with intracranial abnormalities.

## Author Contributions

TL, RR, JP, AG, WP, and GI conceived and designed the study. TL, RR, and JP completed the publication screening, data extraction, and quality assessment of the eligible publications. TL drafted the manuscript, and all authors contributed substantially to its revision. TL takes responsibility for the paper as a whole.

## Conflict of Interest Statement

The authors alone are responsible for the content and writing of the paper. The authors report no competing financial interests.
