# Supplementary material for: A Systematic Review of the Usefulness of Glial Fibrillary Acidic Protein for Predicting Acute Intracranial Lesions following Head Trauma
Source: Front Neurol. 2017 Dec 4;8:652. doi: 10.3389/fneur.2017.00652 (PMC5722790; doi:10.3389/fneur.2017.00652)
Supplement: Supplementary file 1 [file Data_Sheet_1.PDF]

Online Supplementary Material
Appendix 1. Complete literature search protocol

Citation:
Luoto TM, Raj R, Posti JP, Gardner AJ, Panenka WJ, Iverson GI.
A Systematic Review of the Usefulness of Glial Fibrillary Acidic Protein for Predicting Acute Intracranial Lesions following Head Trauma
Frontiers in Neurology. 2017

- Primary searches conducted: 11-12th October 2016
- Update searches conducted: 10th February 2017

Summary of search results

	**Primary search**		**Update search**	
**Database**	**Records identified**	**Records after de-duplication**	**Records identified**	**Records after de-duplication**
CINAHL	39	7	0	0
Cochrane – Other Reviews	1	0	0	0
Cochrane – Trials	17	0	4	0
Embase	1629	1004	64	52
Medline (includes In Process, EPubs)	978	952	21	4
PsycINFO	101	25	2	0
Scopus	1338	254	48	2
Web of Science	436*	171	28	3
**TOTAL**	**4539**	**2413**	**167**	**61**

Database(s): Epub Ahead of Print, In-Process & Other Non-Indexed Citations, Ovid MEDLINE(R) Daily and Ovid MEDLINE(R) 1946 to Present

#	Searches	Results
1	Craniocerebral Trauma/	20788
2	Brain Injuries/	49678
3	Brain Concussion/	5652
4	Diffuse Axonal Injury/	589
5	intracranial hemorrhages/ or exp cerebral hemorrhage/ or exp subarachnoid hemorrhage/	52339
6	Brain Hemorrhage, Traumatic/	199
7	exp Hematoma, Subdural/	8194
8	mtbi.mp.	1642
9	tbi.mp.	17783
10	((head or crani* or capitis or brain* or forebrain* or skull* or hemisphere or intracran* or orbit*) adj5 (injur* or trauma* or lesion* or damage* or wound* or destruction* or oedema* or edema* or fracture* or contusion* or concus* or commotion* or pressur* or laceration* or abnormalit*)).tw.	195493
11	((intracran* or cerebral or crani* or subdural or epidural or subarachnoid or brain or intracerebral) adj5 (h?ematoma* or h?emorrhage*)).tw.	59371
12	concuss*.tw.	5791
13	1 or 2 or 3 or 4 or 5 or 6 or 7 or 8 or 9 or 10 or 11 or 12	288138
14	Glial Fibrillary Acidic Protein/	13866
15	astroprotein.mp.	25
16	glial fibrillary acid* protein*.tw.	13117
17	glial intermediate filament*.mp.	42
18	gfa protein*.mp.	162
19	gfap.mp.	13382
20	14 or 15 or 16 or 17 or 18 or 19	24202
21	13 and 20	2818
22	limit 21 to (english language and yr="1980 -Current")	2726
23	animals/ not (humans/ and animals/)	4292843
**24**	**22 not 23**	**978**

Database(s): Embase Classic+Embase 1947 to 2016 October 10
Search Strategy:

#	Searches	Results	Annotations
1	head injury/	51288	
2	brain injury/ or traumatic brain injury/	122533	
3	concussion/ or brain concussion/	9511	
4	brain contusion/	3318	
5	brain damage/	36184	
6	diffuse axonal injury/	1563	
7	brain hemorrhage/ or subarachnoid hemorrhage/	113109	
8	subarachnoid hemorrhage/	37810	
9	subdural hematoma/	15248	
10	brain hematoma/	7910	
11	mtbi.mp.	2588	
12	tbi.mp.	28336	
13	((head or crani* or capitis or brain* or forebrain* or skull* or hemisphere or intracran* or orbit*) adj5 (injur* or trauma* or lesion* or damage* or wound* or destruction* or oedema* or edema* or fracture* or contusion* or concus* or commotion* or pressur* or laceration* or abnormalit*)).tw.	268471	
14	((intracran* or cerebral or crani* or subdural or epidural or subarachnoid or brain or intracerebral) adj5 (h?ematoma* or h?emorrhage*)).tw.	84252	
15	concuss*.tw.	7849	
16	1 or 2 or 3 or 4 or 5 or 6 or 7 or 8 or 9 or 10 or 11 or 12 or 13 or 14 or 15	460580	
17	glial fibrillary acidic protein/	21577	
18	astroprotein.mp.	27	
19	glial fibrillary acid* protein*.tw.	14839	
20	glial intermediate filament*.mp.	45	
21	gfa protein*.mp.	180	
22	gfap.mp.	18569	
23	17 or 18 or 19 or 20 or 21 or 22	30628	
24	16 and 23	4300	
25	limit 24 to (english language and yr="1980 -Current")	4104	
**26**	**limit 25 to human**	**1629**	

Database(s): PsycINFO 1806 to October Week 1 2016
Search Strategy:

#	Searches	Results
1	traumatic brain injury/	14014
2	brain damage/	17108
3	brain concussion/	1311
4	exp Head Injuries/	5429
5	Diffuse Axonal Injury.mp.	269
6	exp Cerebral Hemorrhage/	1645
7	exp Subarachnoid Hemorrhage/	610
8	mtbi.mp.	1182
9	tbi.mp.	8113
10	((head or crani* or capitis or brain* or forebrain* or skull* or hemisphere or intracran* or orbit*) adj5 (injur* or trauma* or lesion* or damage* or wound* or destruction* or oedema* or edema* or fracture* or contusion* or concus* or commotion* or pressur* or laceration* or abnormalit*)).tw.	56745
11	((intracran* or cerebral or crani* or subdural or epidural or subarachnoid or brain or intracerebral) adj5 (h?ematoma* or h?emorrhage*)).tw.	4038
12	concuss*.tw.	2149
13	1 or 2 or 3 or 4 or 5 or 6 or 7 or 8 or 9 or 10 or 11 or 12	66327
14	glial fibrillary acid* protein*.mp.	1597
15	astroprotein.mp.	0
16	glial intermediate filament*.mp.	1
17	gfa protein*.mp.	0
18	gfap.mp.	1960
19	14 or 15 or 16 or 17 or 18	2593
20	13 and 19	404
**21**	**limit 20 to (human and english language and yr="1980 -Current")**	**101**

CINAHL

#	Query	Results
S1	(MH "Head Injuries") OR (MH "Brain Injuries")	23,095
S2	(MH "Brain Concussion")	2,397
S3	"Diffuse Axonal Injur*"	129
S4	(MH "Intracranial Hemorrhage") OR (MH "Cerebral Hemorrhage+") OR (MH "Hematoma, Epidural") OR (MH "Hematoma, Subdural+") OR (MH "Subarachnoid Hemorrhage")	10,062
S5	mtbi	487
S6	tbi	4,682
S7	TI (((head or crani* or capitis or brain* or forebrain* or skull* or hemisphere or intracran* or orbit*) n5 (injur* or trauma* or lesion* or damage* or wound* or destruction* or oedema* or edema* or fracture* or contusion* or concus* or commotion* or pressur* or laceration* or abnormalit*))) OR AB (((head or crani* or capitis or brain* or forebrain* or skull* or hemisphere or intracran* or orbit*) n5 (injur* or trauma* or lesion* or damage* or wound* or destruction* or oedema* or edema* or fracture* or contusion* or concus* or commotion* or pressur* or laceration* or abnormalit*)))	32,914
S8	TI (((intracran* or cerebral or crani* or subdural or epidural or subarachnoid or brain or intracerebral) n5 (h?ematoma* or h?emorrhage*))) OR AB (((intracran* or cerebral or crani* or subdural or epidural or subarachnoid or brain or intracerebral) n5 (h?ematoma* or h?emorrhage*)))	36,676
S9	TI concuss* OR AB concuss*	2,554
S10	S1 OR S2 OR S3 OR S4 OR S5 OR S6 OR S7 OR S8 OR S9	73,786
S11	glial fibrillary acid* protein*	344
S12	astroprotein	1
S13	glial intermediate filament*	1
S14	gfa protein*	0
S15	gfap	298
S16	S11 OR S12 OR S13 OR S14 OR S15	474
**S17**	**S10 AND S16 (limited to English, Humans, January 1980+)**	**39**

WEB OF SCIENCE

((((head or crani* or capitis or brain* or forebrain* or skull* or hemisphere or intracran* or orbit*) near/5 (injur* or trauma* or lesion* or damage* or wound* or destruction* or oedema* or edema* or fracture* or contusion* or concus* or commotion* or pressur* or laceration* or abnormalit*)) or ((intracran* or cerebral or crani* or subdural or epidural or subarachnoid or brain or intracerebral) near/5 (h?ematoma* or h?emorrhage*)) or "diffuse axonal injur*" or mtbi or tbi or concuss*))*AND* **TOPIC:** ("glial fibrillary acid* protein*" or "glial intermediate filament*" or astroprotein or "gfa protein*" or gfap) *AND* **TOPIC:**(human*)
Timespan: 1980-2016. **Indexes:** SCI-EXPANDED, SSCI, A&HCI, CPCI-S, CPCI-SSH, ESCI.

SCOPUS

TITLE-ABS-KEY (((**head** OR **crani*** OR **capitis** OR **brain*** OR **forebrain*** OR **skull*** OR **hemisphere** OR **intracran*** OR **orbit***) W/5 (**injur*** OR **trauma*** OR **lesion*** OR **damage*** OR **wound*** OR **destruction*** OR **oedema*** OR **edema*** OR **fracture*** OR **contusion*** OR **concus*** OR **commotion*** OR **pressur*** OR **laceration*** OR **abnormalit***)) OR ((**intracran*** OR **cerebral** OR **crani*** OR **subdural** OR **epidural** OR **subarachnoid** OR **brain** OR **intracerebral**) W/5 (**h?ematoma*** OR **h?emorrhage***)) OR **"diffuse axonal injur*"** OR **mtbi** OR **tbi** OR **concuss***) AND TITLE-ABS-KEY (**"glial fibrillary acid* protein*"** OR **"glial intermediate filament*"** OR **astroprotein** OR **"gfa protein*"** OR **gfap**) AND (LIMIT-TO (EXACTKEYWORD , **"Human"**) OR LIMIT-TO (EXACTKEYWORD , **"Humans"**)) AND (LIMIT-TO (LANGUAGE , **"English"**)) AND (LIMIT-TO (PUBYEAR , **2016**) OR LIMIT-TO (PUBYEAR , **2015**) OR LIMIT-TO (PUBYEAR , **2014**) OR LIMIT-TO (PUBYEAR , **2013**) OR LIMIT-TO (PUBYEAR , **2012**) OR LIMIT-TO (PUBYEAR , **2011**) OR LIMIT-TO (PUBYEAR , **2010**) OR LIMIT-TO (PUBYEAR , **2009**) OR LIMIT-TO (PUBYEAR , **2008**) OR LIMIT-TO (PUBYEAR , **2007**) OR LIMIT-TO (PUBYEAR , **2006**) OR LIMIT-TO (PUBYEAR , **2005**) OR LIMIT-TO (PUBYEAR , **2004**) OR LIMIT-TO (PUBYEAR , **2003**) OR LIMIT-TO (PUBYEAR , **2002**) OR LIMIT-TO (PUBYEAR , **2001**) OR LIMIT-TO (PUBYEAR , **2000**) OR LIMIT-TO (PUBYEAR , **1999**) OR LIMIT-TO (PUBYEAR , **1998**) OR LIMIT-TO (PUBYEAR , **1997**) OR LIMIT-TO (PUBYEAR , **1996**) OR LIMIT-TO (PUBYEAR , **1995**) OR LIMIT-TO (PUBYEAR , **1994**) OR LIMIT-TO (PUBYEAR , **1993**) OR LIMIT-TO (PUBYEAR , **1992**) OR LIMIT-TO (PUBYEAR , **1991**) OR LIMIT-TO (PUBYEAR , **1990**) OR LIMIT-TO (PUBYEAR , **1989**) OR LIMIT-TO (PUBYEAR , **1988**) OR LIMIT-TO (PUBYEAR , **1987**) OR LIMIT-TO (PUBYEAR , **1986**) OR LIMIT-TO (PUBYEAR , **1985**) OR LIMIT-TO (PUBYEAR , **1983**) OR LIMIT-TO (PUBYEAR , **1981**))
